# Gene Activation Using FLP Recombinase in *C. elegans*

**DOI:** 10.1371/journal.pgen.1000028

**Published:** 2008-03-21

**Authors:** M. Wayne Davis, J. Jason Morton, Dana Carroll, Erik M. Jorgensen

**Affiliations:** 1 Howard Hughes Medical Institute, University of Utah, Salt Lake City, Utah, United States of America; 2 Department of Biology, University of Utah, Salt Lake City, Utah, United States of America; 3 Department of Biochemistry, University of Utah School of Medicine, Salt Lake City, Utah, United States of America; Stanford University Medical Center, United States of America

## Abstract

The FLP enzyme catalyzes recombination between specific target sequences in DNA. Here we use FLP to temporally and spatially control gene expression in the nematode *C. elegans*. Transcription is blocked by the presence of an “off cassette” between the promoter and the coding region of the desired product. The “off cassette” is composed of a transcriptional terminator flanked by FLP recognition targets (FRT). This sequence can be excised by FLP recombinase to bring together the promoter and the coding region. We have introduced two fluorescent reporters into the system: a red reporter for promoter activity prior to FLP expression and a green reporter for expression of the gene of interest after FLP expression. The constructs are designed using the multisite Gateway system, so that promoters and coding regions can be quickly mixed and matched. We demonstrate that heat-shock-driven FLP recombinase adds temporal control on top of tissue specific expression provided by the transgene promoter. In addition, the temporal switch is permanent, rather than acute, as is usually the case for heat-shock driven transgenes. Finally, FLP expression can be driven by a tissue specific promoter to provide expression in a subset of cells that can only be addressed as the intersection of two available promoters. As a test of the system, we have driven the light chain of tetanus toxin, a protease that cleaves the synaptic vesicle protein synaptobrevin. We show that we can use this to inactivate synaptic transmission in all neurons or a subset of neurons in a FLP-dependent manner.

## Author Summary

Genes turn on and off as a natural part of development. The nematode *C. elegans* has been an important model system for studying the roles of genes in animal development and physiology. However, worm researchers have had a limited toolkit for controlling gene activation. These drawbacks have been particularly restrictive when studying the function of a gene that has different roles in several cell types or at different times in development. Here we describe a way to turn any gene on at a specific time in specific cells. We provide a set of mix-and-match reagents that give researchers a way to quickly build new combinations of regulatory elements. These reagents will allow researchers to express a single gene in a wide array of temporal or spatial patterns, or to serially express many genes in a single cell. As a proof of principle, we made an artificial worm gene composed of a neurotoxin that would block neurotransmission. When we activated the gene in a small number of neurons in adult animals, these cells ceased to function. We anticipate that this new technique will find a wide variety of uses by the *C. elegans* community.

## Introduction

Every widely-used genetic model organism can be manipulated to express genes introduced by the experimenter. In *C. elegans* it is simple to create a transgene by injecting DNA containing a complete genomic region. In most cases the transgene will be expressed in its native temporal and spatial pattern and will rescue the mutant phenotype. However, if the researcher would like to test the function of a gene at a specific time or in a specific tissue of the worm, one would need a very specific promoter. Due to a limited set of promoters available, it is often impossible to express a gene of interest in a specific cell. More importantly, there is only one temporally inducible promoter available – the heat-shock promoter. This promoter has been a workhorse of the field, but has a major drawback because it is expressed ubiquitously. Three techniques have been developed that provide more precise temporal and spatial control by making gene expression dependent on two independently controllable events.

The Chalfie laboratory has developed one solution by expressing the gene product as two complementary halves. In this case, the full gene product is reconstituted only in the cells that express both promoters. When expressed under the control of two different overlapping promoters, the complete protein is only reconstituted in a small number of cells. They have demonstrated that a two-part GFP can be used to label specific cells [1] and that a two-part caspase can be used to kill specific cells [2]. Their technique can also be applied to the temporal control of gene expression. If one of the promoters is a heat-shock inducible promoter, specific cells can be killed on command. The limitation of the two-part system is that it requires gene products that can reconstitute activity from two halves.

A second combinatorial control technique relies on temperature-dependent degradation of the mRNA of the target gene. This method was independently proposed by several groups, but made practical by Getz, Xu and Fire (A. Fire personal communication)[3].The nonsense mediated decay (NMD) pathway specifically degrades mRNAs with long 3′ untranslated regions containing many introns. Transgenes can be engineered with long 3′ UTRs so that their mRNAs are targeted for degradation. Strains with temperature-sensitive mutations in NMD components cannot degrade these mRNAs at the restrictive temperature. Thus, the transgene is more strongly expressed at the restrictive temperature (when the NMD system is not functioning) than at the permissive temperature (when NMD is actively degrading aberrant mRNAs). This system gives some degree of control over expression levels, although there is a moderate background level of expression even in the “off” state, which has limited its use.

Bacaj and Shaham have developed a method to add heat-shock control to a transgene expressed under a tissue-specific promoter[4]. The method uses a mutant background in which the heat-shock response is defective. By rescuing the mutant defect with tissue-specific promoters, a tissue-specific heat shock response is generated. This method requires using the *hsf-1* mutant background and gene expression from the heatshock promoter is still acute. Ideally, a combinatorial expression system would be in the wild-type strain and provide a permanent change in the genotype of the cells of interest.

Here we describe a method that uses FLP recombinase to control transgene expression in *C. elegans*. In this configuration, the transgene is expressed at the combinatorial intersection of two different promoters: either two spatially restricted promoters, or a spatially restricted and temporally controlled promoter.

## Results

### Construct Design

**Figure 1:**
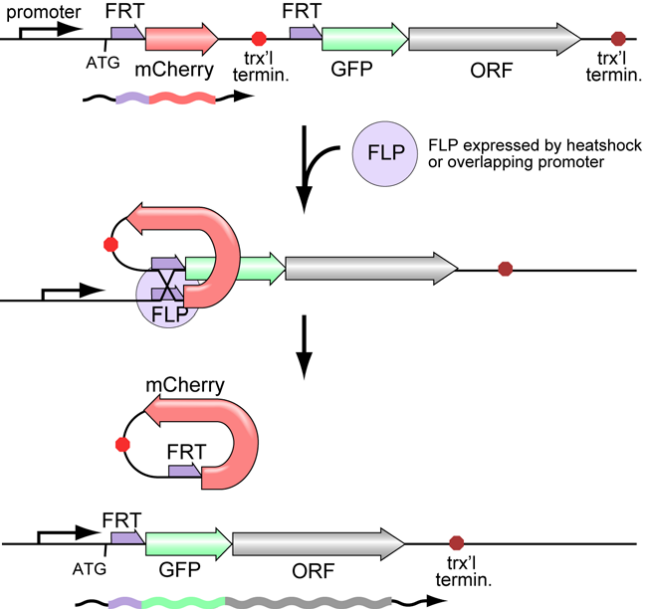
FLP Recombinase Strategy. A FLP inducible transgene in the “off” configuration expresses the mCherry reporter under the control of a promoter sequence. The *let-858* transcriptional terminator prevents transcription into the downstream elements. FLP expression leads to the looping out of the mCherry::*let-858* fragment. This brings the GFP and the in-frame open reading frame (ORF) from the gene of interest under control of the promoter.

The site specific recombinases Cre and FLP have been used in many systems to control gene structure and expression [5]–[10]. These enzymes align tandem copies of the target sequence, perform site-specific recombination, and remove the sequence between the targets as a circular DNA molecule. If the intervening sequence disrupts expression, removal by recombination will allow the transgene to be activated. We designed an “off cassette”, composed of a putative transcriptional terminator, that could be placed between a promoter and a coding region to disrupt expression of the coding region (Figure 1). Expression of FLP recombinase will excise the cassette as a circular DNA molecule. This rearrangement will place the promoter adjacent to the downstream coding regions, converting the transgene to the “on” state. Thus, expression is dependent on both the promoter driving the coding region and the promoter driving expression of FLP.

The constructs are designed to provide a fluorescent readout in either the ‘off’ or ‘on’ state. The FRT-flanked “off cassette” contains the mCherry coding sequence followed by the 3′ genomic region from the *let-858* gene. The red-fluorescent mCherry protein acts as a reporter for the promoter activity of the transgene, verifying that the transgene is present and expressing in the expected cell types prior to FLP-induced recombination. The *let-858* 3′ genomic region provides the poly-adenylation signal for mCherry mRNA as well as a putative transcriptional terminator, preventing expression of downstream sequences. GFP acts as a reporter for transcriptional read-through or reinitiation of transcription in the *let-858* genomic region. Recombination of the FRT sequences removes the mCherry coding sequence and the terminator and brings a GFP coding region under control of the promoter, to indicate that the FLP reaction was successful. The coding region for any gene of interest can be fused in frame to the 3′ end of the GFP sequence, thus providing FLP-inducible expression of that protein.

### Modular Design

**Figure 2:**
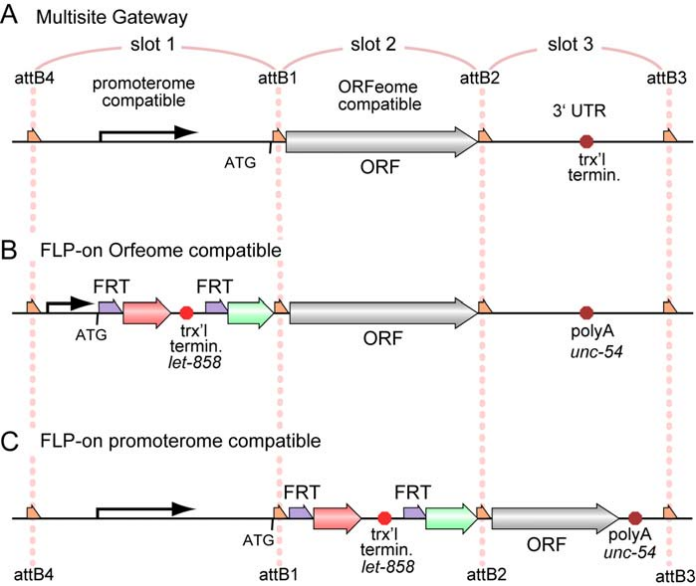
The Basic Design of FLP-Dependent Transgenes Using Gateway. A standard multisite Gateway construct (A) is composed of a promoter, open reading frame (ORF) and 3′ untranslated region (UTR) that terminates transcription. These can be made FLP inducible by adding the FRT flanked cassette in slot 1 flanked by the attB4 and attB1 lambda recombination targets(B), or in slot 2 flanked by the attB1 and attB4 lambda recombination sites (C). These two formats allow compatibility with the already constructed promoterome or ORFeome clones. The red and green arrows represent mCherry and GFP coding regions, as in Figure 1. Red octagons represent the polyadenylation site and transcription terminator from the *let-858* 3′ genomic DNA or the polyadenylation site from the *unc-54* 3′ genomic region.

To speed assembly of constructs and to make use of genome reagents generated by other laboratories, we based our constructs on the Multisite Gateway™ *in vitro* recombination system from Invitrogen [11],[12]. These vectors allow rapid, modular construction of plasmids using a site-specific recombinase from the bacteriophage lambda (Figure 2A). The recombinase target sequences are designed to allow pairwise recombination between specific DNA sequences. The standard multisite system uses three libraries of entry vectors: a promoter library, a cDNA library, and transcriptional terminator library (to be recombined into slots 1, 2 or 3, respectively). Individual components from these libraries can be selected and mixed with a destination vector to generate a desired combination of promoter, cDNA and terminator in a single reaction. Many reagents are already available for the construction of *C. elegans* expression constructs using this system. The *C. elegans* promoterome consortium has cloned the 5′ regulatory regions of approximately 6,000 different *C. elegans* genes into Gateway ‘entry’ vectors compatible with the slot 1 entry vectors [13]. The ORFeome project from the Vidal laboratory has cloned cDNAs from approximately 11,000 genes into slot 2 entry vectors [14].

Because the promoterome constructs are designed to recombine directly to ORFeome constructs, it was not possible to introduce the off-cassette between them. Instead, we built vectors that have the off-cassette in either the promoter slot (slot 1) (Figure 2B) or in the cDNA slot (slot 2) (Figure 2C). The former arrangement requires construction of a promoter-FRT construct, but is compatible with the existing ORFeome library. The latter requires placing the ORF to the third slot, but is compatible with the existing promoterome library.

The ORFeome-compatible format requires that the “off-cassette” be cloned into entry vectors containing various promoters. We generated several potentially useful promoter-“off-cassette” entry clones (Figure 2B, Table 1). These plasmids can then be recombined with any ORFeome clone to produce a large number of different open reading frames under the same FLP-inducible promoter sequence. This arrangement is particularly useful when one is primarily interested in expressing a number of different proteins in a particular cell or tissue.

**Table 1:** Orfeome Compatible Constructs.

Type	Gene elements	Application	Plasmid name
Promoter (Slot 1)	*Punc-47*::FTF	GABA neuron expression	pWD179
	*Prab-3*:: FTF	Pan-neuronal expression	pWD180
	*Pmyo-2*:: FTF	Pharyngeal muscle expression	pWD177
ORF (Slot 2)	tetanus toxin	Neurotransmission inactivation	pWD157
	HIS-11	Nuclear labeling	pWD195
UTR (Slot 3)	*unc-54* UTR	Polyadenylation site	pMH473

FTF = FRT::mCherry::*let-858* 3′ Terminator:: FRT.

For the promoterome-compatible constructs, we placed the off-cassette into the position usually occupied by the ORF of interest. We then used standard cloning methods to place several ORFs of interest into slot 3 entry vectors (Figure 2C, Table 2). Any of the set of promoterome clones can be recombined with these clones to express a single ORF in a wide array of tissues in a FLP-dependent manner. This arrangement is particularly useful when one is determining the focus of gene rescue or inactivating different groups of cells.

**Table 2:** Promoterome Compatible Constructs.

Type	Gene elements	Application	Plasmid name
ORF (Slot 2)	FTF:GFP	FRT flanked terminator cassette	pWD178
UTR (Slot 3)	HIS-11::*unc-54* UTR	Nuclear labeling	pGH42
	tetanus toxin::*unc-54* UTR	Neurotransmission inactivation	pWD170
	Caspase-3(C)::*unc-54* UTR	Cell ablation. Requires complementary caspase-3 fragments.	pWD203
	Caspase-3(N)::*unc-54* UTR		pWD204

### FLP-Mediated Recombination

**Figure 3:**
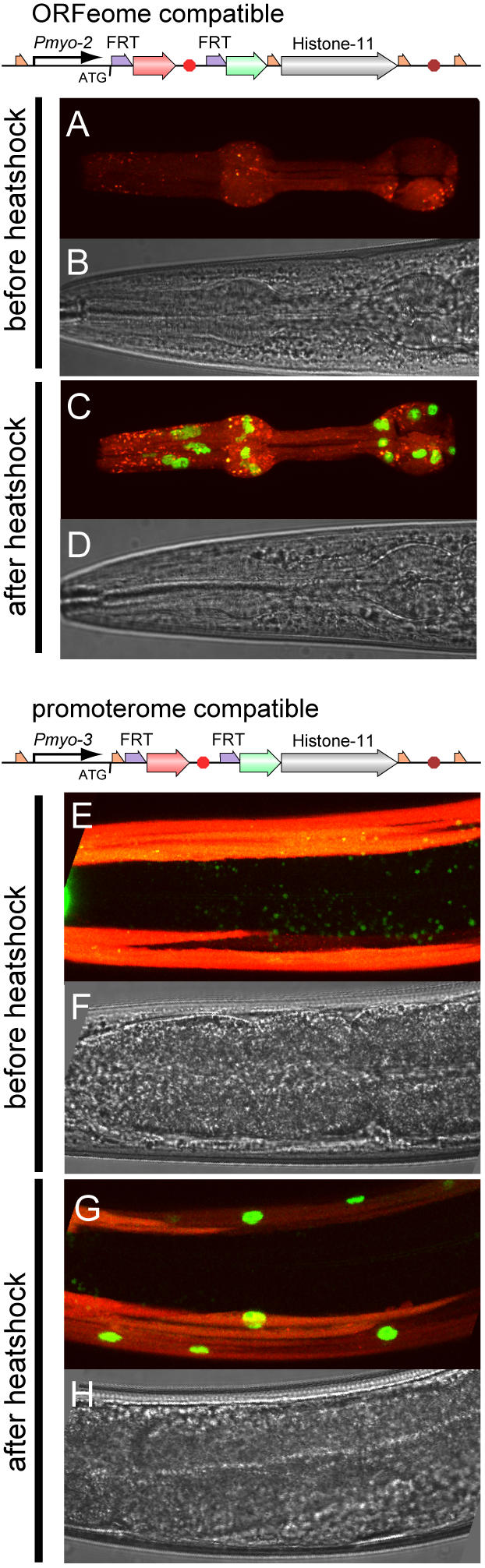
FLP-Dependent GFP-Histone Expression. Before heat induction, the *myo-2* promoter (A,B in strain EG4866 pWD200) or *myo-3* promoter (E,F in strain EG4859 pWD198) drive mCherry expression in the pharyngeal or body muscle, respectively. FLP recombinase is induced by activation of the heatshock promoter; FLP removes the mCherry terminator. 15 hours after heat induction, these transgenes produce nuclear-localized GFP-histone fusion protein (C,D,G, H).

As a proof of principle, we produced two FLP-inducible constructs that would express GFP-tagged histone in different muscle cells. The first construct was in the ORFeome compatible configuration described in Figure 2B. We used a pharyngeal muscle promoter (*Pmyo-2*) followed by the “off-cassette” in the first Gateway slot. We added the HIS-11 open reading frame in the second slot and an *unc-54* 3′ polyadenylation site in the third slot (Figure 3A–D). We injected this plasmid together with a plasmid encoding FLP-recombinase under the control of the *hsp-16.48* heat shock promoter [15] and a *lin-15*(+) co-injection marker to produce a line of transgenic worms. As expected, these worms expressed diffuse red fluorescence in their pharyngeal muscles with no apparent GFP fluorescence (Figure 3A, B). The lack of GFP fluorescence confirms that the *let-858* 3′ genomic region functions as expected to prevent read-through into the downstream gene. We then exposed the worms to a one hour, 34° heat shock and imaged the worms 2 hours, 3 hours and 15 hours later. Although no GFP was apparent at 2 hours, the heat shock-induced expression of the GFP::HIS-11 fusion protein was visible at three hours (Figure S1) and was strong at 15 hours (Figure 3C, D). From roughly 200 animals observed under a dissecting microscope, every worm examined exhibited green nuclei in muscle cells expressing mCherry. We counted the GFP positive nuclei in five randomly selected adult worms 22 hours after a 34° heat shock. We found strong expression in 98% (123/125) of major pharyngeal muscle cells (pm3-pm8, 25 nuclei per worm). Determining expression in pm1 and pm2 is complicated, since these cells are surrounded by the pm3 muscle cell (see http://www.wormatlas.org/handbook/fig.s/alim9.jpg). Bright mCherry fluorescence obscured expression of the cassette in these cells before heatshock. Thus, we could not determine whether the *myo-2* promoter was driving expression of the transgene. After heatshock we did not score GFP expression in any pm1 nuclei (0/15) and only identified weak expression in 7/15 pm2 nuclei. In summary, robust expression due to recombination was observed in all cells in which the transgene was unequivocally expressed.

The second construct was in the promoterome-compatible configuration described in Figure 2C. We generated this construct by recombining a *myo-3* promoterome plasmid in the first position, the FRT cassette was placed into the second position and a HIS-11 ORF cloned in front of the *unc-54* 3′ genomic region in the third position. This construct should express the GFP::HIS-11 fusion in the body muscles of the worm after FLP expression. There was diffuse red but no green fluorescence in the body wall muscles prior to heat shock (Figure 3E, F), but strong induction of nuclear-localized GFP 15 hours after a 1 hour exposure to 34° heat shock (Figure 3G, H). Using a dissection microscope to survey a large number of animals, GFP was detected in body muscle nuclei in all transgenic animals. We examined 5 animals in greater detail and observed expression in body wall muscles, vulval muscles and the anal depressor muscles in all animals, demonstrating that FLP works in all major body muscle types.

If all of the copies of the transgene are recombined, mCherry expression should not occur after FLP expression. However, in all cells examined (using the *myo-2*, *myo-3* and *unc-47* promoters), we never saw substantial loss of mCherry expression even after induction of the GFP fusion protein. Although the half-life of mCherry in worm cells is not known, the half life of GFP in body muscle cells is greater than 24 hours [16]. Thus, perdurance of mCherry could be a source of this remaining expression. It is also possible that several copies of the transgene on the extrachromosomal array are refractory to recombination. This could be due to the chromatin structure of the repetitive arrays, or damage to the arrays that occurred during their formation.

### Applications

One important application for the FLP-on method is to silence neurotransmission in specific neurons in a temporally-controlled manner. The tetanus toxin light chain is a highly specific protease that recognizes and cleaves synaptobrevin [17]. Since synaptobrevin is one of the three SNARE-class proteins required for calcium-mediated release of neurotransmitter [18], expressing tetanus toxin eliminates the ability of a neuron to signal through chemical synapses. The high specificity of tetanus toxin preserves all other functions of the neuron, including electrical coupling through gap junctions. Temporal control of tetanus toxin expression is important for two reasons. First, synaptic transmission is essential for development. Animals lacking acetylcholine neurotransmission (in *cha-1* mutants) or all synaptic neurotransmission (in *unc-13* deletion alleles) are arrested in the first larval stage. Thus, loss of synaptic transmission in at least some neurons may lead to broad developmental defects that blind the investigator to functions for a neuron in the adult. Using the FLP-on method, expression of tetanus toxin can be activated after the developmental requirement for a neuron. Conversely, early loss of neuronal function can lead to developmental compensation in the nervous system. For example, when particular sensory neurons were ablated in males in the L3 stage the nervous system compensated for their loss; by contrast ablation of these same neurons in the L4 stage led to behavioral abnormalities in the adult [19]. The ability to silence these neurons in adulthood allows one to assay the function of the synaptic connectivity of a neuron in an existing developmentally-normal circuit.

To test if tetanus toxin induction could inactivate neurotransmission, we designed an ORFeome-compatible tetanus toxin expression construct. The tetanus toxin sequence was inserted into slot 2, and the FRT-flanked terminator was placed in slot 1 after the GABA-specific neuron promoter *Punc-47*. *unc-47* encodes the vesicular GABA transporter required to fill synaptic vesicles with GABA, and is expressed in all GABA neurons. We chose this promoter because loss of GABA function produces two distinct phenotypes: a locomotory phenotype and a defecation motor program defect.

Animals lacking GABA neurotransmission exhibit a distinctive locomotory defect [20]. These animals cannot back when touched on the head but rather execute an accordion-like “shrinking” response. This symmetrical contraction of the body muscles occurs due to the lack of contralateral inhibitory inputs from the GABA motor neurons. Prior to heat shock, transgenic worms exhibited normal movement. Animals were exposed to heat shock at 34° for one hour. 24 hours later the animals exhibited a clear shrinking phenotype (Video S1). The structure of the GABA neurons was not affected by toxin expression (Figure S2), suggesting that the toxin was simply silencing synaptic transmission in these neurons. As expected the shrinking phenotype was associated with the presence of the transgene: 99 of 100 shrinker animals carried the P*myo-2*::GFP extrachromosomal array marker. The presence of nonshrinking animals in the population was due to loss of the extrachromosomal array: 29 of the 30 non-shrinker animals had lost the P*myo-2*::GFP transgene marker. The one non-shrinking animal carrying the array lacked the mCherry marker in the VD and DD motor neurons, demonstrating that this animal was a somatic mosaic which lacked the array in the motor neurons.

**Figure 4:**
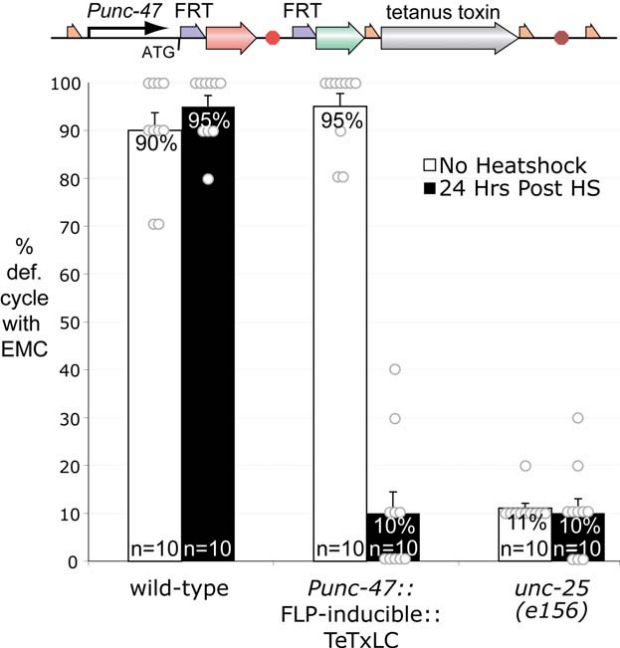
FLP-Dependent Tetanus Toxin Expression. A GFP-tetanus toxin fusion product was expressed from the GABA neuron specific *unc-47* promoter in a FLP-dependent manner. *C. elegans* adult hermaphrodites undergo a defecation cycle every 50 seconds. Wild-type worms execute enteric muscle contractions (EMCs) during 90% of defecation cycles. Prior to heat shock, the FLP-inducible GFP-tetanus toxin animals are not significantly different from the wild type (95%). However, after heat shock they exhibit enteric muscle contractions in only 10% of defecation cycles, significantly less than the wild type and not significantly different from *unc-25* mutants. *unc-25* encodes the biosynthetic enzyme for GABA, glutamic acid decarboxylase. Similar to animals in which tetanus toxin is blocking synaptic transmission from the GABA neurons, *unc-25* mutants exhibit enteric muscle contractions in only 11% of defecation cycles. Neither wild-type nor *unc-25* worms are significantly affected 24 hours after heat shock (95% and 10% EMC/defecation cycle). Ten defecation cycles were scored for each of ten worms for each condition. Counts were done between 23 and 27 hours after heat shock. Error bars represent the standard error of the mean.

GABA function is also required for the motor program of the defecation cycle [21],[22]. The defecation motor program requires the AVL and DVB GABA neurons to stimulate contraction of the enteric muscles via a GABA-gated cation channel [23]. Prior to heat shock, transgenic worms expressing mCherry in the AVL and DVB GABA neurons had wild-type enteric muscle contractions during the defecation cycle (n = 10 worms, 10 cycles per worm, Figure 4). After heat shock, transgenic animals lacked the enteric muscle contractions during the defecation cycle (Figure 4), as expected for loss of GABA neurotransmission. Because AVL and DVB are partially redundant, these data suggest that FLP function must be greater than 95% effective. In addition, we were able to see induction of GFP-tagged tetanus toxin by confocal microscopy (not shown). Tetanus toxin expression is continuous using this FLP-on construct; thus, unlike direct heatshock-induced expression of the toxin, the behavioral change is permanent.

## Discussion

FLP-dependent gene expression will have two general uses in *C. elegans*: to provide spatial specificity and temporal specificity for gene expression. Because it provides expression within the spatial overlap of two promoters, the method essentially squares the number of transgene expression patterns now available. In many cases, especially in the nervous system, this can restrict expression to single cells of the worm.

FLP recombinase can also be used to provide temporal specificity for gene expression. For most purposes temporal control is best provided by activation of the heatshock promoter. Thus, heatshock-driven expression of FLP recombinase will confer temporal specificity to any promoter. This will be particularly useful for expression of dominant negative or constitutively activated gene products that may kill cells before their effects can be assayed. Moreover, acute expression can outflank the criticism most often thrown at genetic analysis –that homeostatic mechanisms will compensate for chronic genetic changes. In this way the heatshock FLP-on method and the cell-specific rescue of *hsf-1* method developed by the Shaham laboratory are similar. In the FLP-on method, expression is permanently activated, whereas in the *hsf-1* rescue method, expression is acute and depends on the length of the heatshock response. Depending on the circumstances and the gene product being expressed, one method may be more advantageous than the other.

For the neuroscientist, FLP-on constructs provide a method for analyzing the role of a neuron in a circuit by killing a specific cell, inactivating the cell, or activating the cell. By combining our system with the Chalfie split caspase system (Table 1), cells can be killed at the intersection of three promoters. These three promoters could each provide a spatial component, giving extremely tight spatial control to potentially address the few single neuron types that have escaped the two-promoter system. Alternatively, one of the three could provide temporal inducibility, adding heat shock control onto many two-promoter single-cell killing experiments. Silencing the cell can allow specific dissection of the chemical synapses present in the system while leaving the gap junction connections in the network intact. In addition, leaving the cell in place will minimize any developmental perturbations of the circuit that might be caused by removing a neuron by the cell death pathway. In addition to eliminating a cell entirely, or silencing the chemical neurotransmission in a cell, single cells can be electrically silenced or activated on command by expressing halorhodopsin, a light activated chloride pump protein [24], or channelrhodopsin, a light activated cation channel protein [25], at the intersection of two spatial promoters.

For the developmental biologist, FLP-on constructs can provide temporal control of gene expression so that the role of a gene during different developmental periods can be evaluated. This application is limited by the time delay required for FLP expression, recombination and gene expression. We observed a three hour delay from the end of heat shock to the expression of P*myo-2*::GFP. FLP regulation will also be useful for analyzing promoter expression patterns by permanently marking descendents of cells that have expressed a promoter. Combined with the known cell lineage of *C. elegans*, the expression pattern can quickly pinpoint the expression pattern of a transgene that may come on very briefly and in only a few cells in the embryo. Using traditional GFP reporters, such expression might be missed entirely, or if it is detected it might be very difficult to unambiguously identify the expressing cell among the dividing cells of the embryo. This technique could be combined with forward genetic screens. A FLP-dependent histone GFP reporter (Table 1) will easily identify mutant backgrounds in which a gene is transiently misexpressed during development.

In conclusion, FLP-dependent excision of a transcriptional terminator provides a simple way to make expression of a transgene dependent on the activity of two promoters. Depending on the promoters used, FLP-on constructs can confer combinatorial spatial or temporal control of gene expression in *C. elegans*. We anticipate that the combination of the wide availability of the Gateway reagents and the imagination of the *C. elegans* community will yield many new applications.

## Materials and Methods

### Plasmids

pWD157 (slot 2 TeTx): Tetanus toxin light chain was PCR amplified from CMV-LC-Tx (Heiner Niemann) using primers containing attB1 and attB2 tails GGGGACAAGTTTGTACAAAAAAGCAGGCTTAATGCCGATCACCATCAACAACTTC and GGGGACCACTTTGTACAAGAAAGCTGGGTTTAAGCGGTACGGTTGTACAGGTT and recombined with the attP1 and attP2 sites in the slot 2 donor vector pDONR221 (Invitrogen) using the BP recombination reaction.

pWD170 (slot 3 TeTx): Tetanus toxin light chain was PCR amplified from CMV-LC-Tx using primers GTATGCCGATCACCATCAACAAC and TTAAGCGGTACGGTTGTACAGG and cloned as a blunt fragment into pMH472 using SrfI. pMH472 is a slot 3 entry vector containing a SrfI site followed by two stop codons and then the *unc-54* 3′ UTR.

pPD119FRTRFPGFP: A fragment of mRFP1 was PCR amplified and cloned between the SalI and SmaI sites in pPD118.33. pPD118.33 is a *Pmyo-2::GFP* plasmid (gift of Andrew Fire). Tandem FRT sites [8] were cloned at the junctions as SalI-BamHI and MluI-SmaI double-stranded oligonucleotides.

pWD176 (*Pmyo-2*-FRT-mCherry-terminator-FRT-GFP-terminator): pPD119FRTRFPGFP was modified to replace mRFP with mCherry. An MluI-KpnI double-stranded oligonucleotide containing an FRT fragment in a different frame was used to replace the second FRT in pPD119FRTRFPGFP. The plasmid was then cut with BamHI and Mlu and a PCR fragment containing mCherry followed by the *let-858* 3′ end was ligated in as a BamHI-BssHII digested fragment. This produced a *myo-2* promoter::FRT::mCherry terminator::FRT::GFP::*unc-54* 3′ UTR plasmid.

pWD177 (slot 1 *Pmyo-2*-FRT-mCherry-terminator-FRT): The *Pmyo-2*::mRFP FRT cassette::GFP fragment from pPD119FRTRFPGFP was PCR amplified using primers containing attB4 and attB1 tails GGGGACAACTTTGTATAGAAAAGTTGCTTGCATGCCTGCAGGTCGAGG and GGGGACTGCTTTTTTGTACAAACTTGTTTTGTATAGTTCGTCCATGCCATG and recombined with the attP4 and attP1 sites in the slot 1 donor vector pDONR P4-P1R (Invitrogen) using the BP recombination reaction to make pWD159. The mRFP cassette was replaced with mCherry using a BamHI-XhoI fragment from pWD176. This plasmid was sequenced with primers T7, M13fwd, mCh r1: CTTTCACTTGAAGCTTCCCATCCC, GFP-I-r2: CTCCAGTGAAAAGTTCTTCTCC, and GFP-I-r1: TTGTGCCCATTAACATCACC.

pWD178 (slot 2 FRT-mCherry-terminator-FRT): The mRFP FRT cassette::GFP fragment from pPD119FRTRFPGFP was PCR amplified using primers containing attB1 and attB2 tails GGGGACAAGTTTGTACAAAAAAGCAGGCTTACGAAGTTCCTATTCTCTAGA and GGGGACCACTTTGTACAAGAAAGCTGGGTTTTTGTATAGTTCGTCCATGCC and recombined with the attP1 and attP2 sites in pDONR221 (Invitrogen) using the BP recombination reaction. The mRFP cassette was replaced with mCherry using a BamHI-XhoI fragment from pWD176. This plasmid was sequenced with primers T7, M13fwd, GFP-I-r2: CTCCAGTGAAAAGTTCTTCTCC, and GFP-I-r1: TTGTGCCCATTAACATCACC

pWD179: The 1.2 kb *unc-47* promoter was PCR amplified from plasmid pKS4.1 (K. Schuske) using primers: CGAACGCATGCGGATCCCGGAACAGTCGAAAG and CGAACGTCGACGCATCTGTAATGAAATAAATGTGACGCTG. This sequence was inserted into pWD159 as an Sph-Sal fragment. The mRFP cassette was replaced with mCherry using a SalI-BstZ17I fragment from pWD176. This plasmid was sequenced with primers T7, M13fwd, mCh r1: CTTTCACTTGAAGCTTCCCATCCC, GFP-I-r2: CTCCAGTGAAAAGTTCTTCTCC, and GFP-I-r1: TTGTGCCCATTAACATCACC.

pWD180: The 1.2 kb *rab-3* promoter was PCR amplified from N2 genomic DNA using primers: CGAACGCATGCATCTTCAGATGGGAGCAGTGG and CGAACGTCGACGCATCTGAAAATAGGGCTACTGTAGAT. This DNA was inserted into pWD159 as an Sph-Sal fragment. The mRFP cassette was replaced with mCherry using a BamHI-XhoI fragment from pWD176. This plasmid was sequenced with primers T7, M13fwd, mCh r1: CTTTCACTTGAAGCTTCCCATCCC, GFP-I-r2: CTCCAGTGAAAAGTTCTTCTCC, and GFP-I-r1: TTGTGCCCATTAACATCACC.

pWD195 (slot 2 Histone 2B): The *his-11* ORF was PCR amplified using primers containing attB1 and attB2 tails GGGGACAAGTTTGTACAAAAAAGCAGGCTTACCACCAAAGCCATCTGCCAAGG and GGGGACCACTTTGTACAAGAAAGCTGGGTATTACTTGCTGGAAGTGTACTTGG using pGH42 (G. Hollopeter) as template. The DNA fragment was recombined with the attP1 and attP2 sites in pDONR221 (Invitrogen) using the BP recombination reaction.

pWD198 (*Pmyo-3*-FRT-mCherry-FRT-GFP-Histone): A multisite LR reaction was performed using pEntry[4-1] *Pmyo-3* (Open Biosystems), pWD178, pGH42 (G. Hollopeter), and pDEST R4-R3 (Invitrogen).

pWD199 (*Punc-47*-FRT-mCherry-FRT-GFP-TeTx): A multisite LR reaction was performed using pWD179, pWD157, pMH473 (M. Hammarlund), and pDEST R4-R3 (Invitrogen). pMH473 is an attP2-attP3 entry clone carrying the *unc-54* 3′ polyadenylation site.

pWD200 (*Pmyo-2*-FRT-mCherry-FRT-GFP-Histone): A multisite LR reaction was performed using pWD177, pWD195, pMH473 and pDEST R4-R3 (Invitrogen).

pWD203 (slot 3 Caspase C-terminus): The caspase 3 C-terminal fragment, leucine zipper and *unc-54* 3′ UTR from TU#813 [2] was PCR amplified using primers: GGGGACAGCTTTCTTGTACAAAGTGGGAAGTGGTGTTGATGATGACATGGCG and GGGGACAACTTTGTATAATAAAGTTGCCATAGACACTACTCCACTTTC and BP cloned into pDONR P2R-P3 (Invitrogen).

pWD204 (slot 3 Caspase N-terminus) The caspase 3 N-terminal fragment, leucine zipper and *unc-54* 3′ UTR from TU#814 [2] was PCR amplified using primers containing attB2 and attB3 tails: GGGGACAGCTTTCTTGTACAAAGTGGGAATGGCTAGCGCACAGCTGGAGAAG and GGGGACAACTTTGTATAATAAAGTTGCCATAGACACTACTCCACTTTC and BP cloned into pDONR P2R-P3 (Invitrogen).

pWD79-2RV (*Phsp-16-48*:FLP): A PCR fragment containing the FLP coding sequence from pOG44 (Stratagene) was cloned as an MluI-NheI fragment into pJL44 (J.L. Bessereau). pJL44 contains the *Phsp-16-48* heat-shock promoter and the *glh-2* 3′ UTR. The FLP coding sequence in pOG44 contains a point mutation which was repaired using a PCR fragment from the FLP coding sequence cloned into pBR322 (Makkuni Jayaram). An artificial intron was introduced into the FLP coding sequence at the EcoRV site using a double-stranded oligo: GTAAGTTTAAACATATATACTAACTAACCCTGATTATTTAAATTTTCAG.

pWD172 (slot 2 FLP-stop): The FLP ORF was PCR amplified from pWD79-2RV using primers containing attB1 and attB2 tails: GGGGACAAGTTTGTACAAAAAAGCAGGCTTAATGCCACAATTTGGTATATTATGT and GGGGACCACTTTGTACAAGAAAGCTGGGTATTATATGCGTCTATTTATGTAGGATG and BP cloned into pDONR221 (Invitrogen). This version of FLP contains a stop codon at the native C terminus.

pWD173 (slot 2 FLP-no stop): The FLP ORF was PCR amplified from pWD79-2RV using primers containing attB2 and attB3 tails: GGGGACAAGTTTGTACAAAAAAGCAGGCTTAATGCCACAATTTGGTATATTATGT and GGGGACCACTTTGTACAAGAAAGCTGGGTATATGCGTCTATTTATGTAGGATG and BP cloned into pDONR221 (Invitrogen). This version of FLP does not contain a stop codon, and can be used to make C-terminally tagged proteins.

Gateway BP and LR in vitro recombination reactions were carried out according to manufacturer instructions.

### Strains

Strains used in this study: The wild type is Bristol N2.

EG3251 *unc-25(e156) III*.

EG4859 *oxEx1099* was made by injecting: pWD198 (*Pmyo-3*-FTF-GFP-Histone) at 5 ng/ul, pWD79-2RV (*Phsp-16-48*:FLP) at 45 ng/ul, pPD118.33 (*Pmyo-2*::GFP)(A. Fire) at 1 ng/ul, and pL15EK (*lin-15(+)*)[26] at 50 ng/ul into N2 animals.

EG4860 *oxEx1100* was made by injecting: pWD199 (*Punc-47*-FTF-GFP-TeTx) at 5 ng/ul, pWD79-2RV (*Phsp-16-48*:FLP) at 45 ng/ul, pPD118.33 (*Pmyo-2*::GFP)(A. Fire) at 1 ng/ul, pL15EK (*lin-15(+)*)[26] at 50 ng/ul into N2 animals.

EG4866 *lin-15(n765ts) X oxEx1101* was made by injecting: pWD200 (*Pmyo-2*-FTF-GFP-Histone)at 2 ng/ul, pWD79-2RV (*Phsp-16-48*:FLP) at 45 ng/ul, pL15EK (*lin-15(+)*)[26] at 50 ng/ul into MT1642 *lin-15(n765ts)*.

‘FTF’ symbolizes the off cassette composed of FRT-mCherry-terminator-FRT.

## Supporting Information

Figure S1 FLP-Dependent GFP-Histone Expression. Before heat induction, the myo-2 promoter drives mCherry expression in the pharyngeal muscle. FLP recombinase is induced by a 34° heat shock for one hour. Three hours after heat induction, the transgene produces nuclear-localized GFP-histone fusion protein.
(0.18 MB TIF)

Figure S2 The GABA Nervous System 23 Hours after Heatshock. A transgenic strain carrying FLP inducible expression of tetanus toxin (EG4860) was heat shocked one hour at 34°. 23 hours later, mCherry in the GABA nervous system was imaged. The architecture of the GABA nervous system is normal; thus heat shock, FLP expression, and tetanus toxin does not affect the anatomy of the neurons. Anterior is to the left, dorsal is at the top.
(0.07 MB TIF)

Video S1 A transgenic strain carrying FLP inducible expression of tetanus toxin before and after heat shock. An unc-25 mutant that lacks GABA synthesis is shown for comparison.
(0.62 MB MPG)
