# Supplementary figures and images for: Gene Activation Using FLP Recombinase in C. elegans
Source: PLoS Genet. 2008 Mar 21;4(3):e1000028. doi: 10.1371/journal.pgen.1000028 (PMC2265415; doi:10.1371/journal.pgen.1000028)

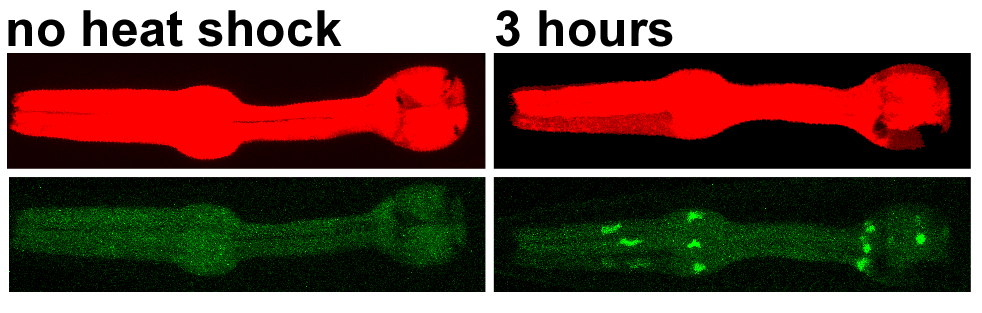

Supplement: Figure S1 — FLP-Dependent GFP-Histone Expression. Before heat induction, the myo-2 promoter drives mCherry expression in the pharyngeal muscle. FLP recombinase is induced by a 34° heat shock for one hour. Three hours after heat induction, the transgene produces nuclear-localized GFP-histone fusion protein. (0.18 MB TIF) [file pgen.1000028.s001.tif]

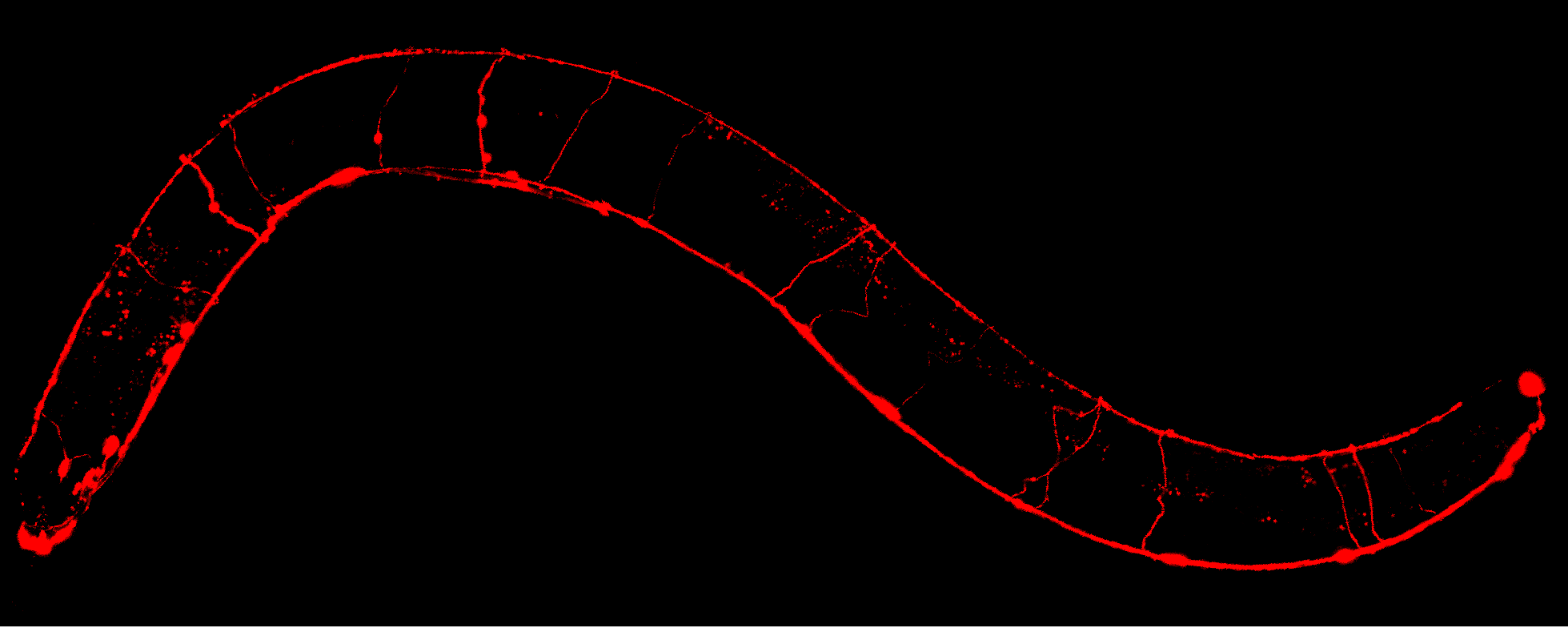

Supplement: Figure S2 — The GABA Nervous System 23 Hours after Heatshock. A transgenic strain carrying FLP inducible expression of tetanus toxin (EG4860) was heat shocked one hour at 34°. 23 hours later, mCherry in the GABA nervous system was imaged. The architecture of the GABA nervous system is normal; thus heat shock, FLP expression, and tetanus toxin does not affect the anatomy of the neurons. Anterior is to the left, dorsal is at the top. (0.07 MB TIF) [file pgen.1000028.s002.tif]
